# Exploring challenges in later-life relationships: a qualitative study of the queries posted on counselling websites

**DOI:** 10.3389/fpsyg.2023.1245736

**Published:** 2023-10-31

**Authors:** Stanislav Svačinka, Anna Ševčíková, Jaroslav Gottfried

**Affiliations:** ^1^Psychology Research Institute, Faculty of Social Studies, Masaryk University, Brno, Czechia; ^2^Department of Psychology, Faculty of Education, University of South Bohemia in České Budějovice, České Budějovice, South Bohemia, Czechia

**Keywords:** later-life relationships, online counselling, partnership problems, ageing, qualitative study

## Abstract

**Introduction:**

Divorce rates among individuals aged 50 and above are on the rise. Given the greater life expectancy compared to previous generations, this is an issue that is affecting an increasing number of people. Therefore, it warrants an inquiry into the challenges these individuals encounter in their intimate relationships.

**Methods:**

This study analyzed 225 relationship-related queries posted on Czech counselling websites to identify the strains and stressor patterns that older adults face in their relationships. The queries were limited to those that concerned themes and problems related to partnerships, were posted by one of the partners aged 60 or over, and were analyzed using inductive thematic analysis.

**Results:**

Four main relationship issues were identified: infidelity and jealousy; relationship estrangement and cooling; undesirable changes in personality; and illness and somatic issues. Additionally, three recurring themes were identified that made the problems more demanding and that were specific to older age: lack of norms for relationships in that age group, absence of resources to tackle the issues, and personal calculation for Time Spent and Time Remaining.

**Discussion:**

The research found that the types of problems encountered by older adults were similar to those experienced by younger individuals. However, the way these problems were perceived and processed was influenced by specific aspects of aging, such as societal expectations, available coping resources, and the perception of time. The findings also highlighted the challenges faced by older adults in terms of relationship norms, sexual functioning, and personality changes.

## 1. Introduction

Relationships at older age have unique characteristics. First, the duration of these relationships can be extended due to increasing life expectancy, which has significantly improved over the past 30 years (WHO, 2023). Second, this substantial improvement in life expectancy has also given rise to a generation of older people who have no comparable experience with long-term relationships in a prolonged age. Unlike prior birth cohorts, in which partnerships tended to collapse relatively soon after retirement, primarily due to the male partner’s death, this generational shift is reshaping the landscape of later-life relationships (CZSO, 2018). Third, recent research has pointed to an increase in divorce rates among generations in their 50 s and older across different countries (Brown and Lin, 2012; Kennedy and Ruggles, 2014; CZSO, 2020). Fourth, establishing new relationships at older age proves to be challenging because the chance to re-partner decreases with age (Vespa, 2012; Brown et al., 2019). This generational shift, combined with rising divorce rates and difficulties in re-partnering, underscores the need to investigate the specific issues and conflicts that individuals from this generation are experiencing in their intimate relationships. Therefore, this study focuses on the challenges and strains that couples face in their later-life relationships that prompt searches for help on counselling websites.

There has been a consensus in the past that later-life relationships are characterized by fewer marital conflicts and relatively high marital satisfaction (Ward, 1993; Birditt et al., 2012), mainly thanks to older people’s tendency to adopt strategies that result in less negative emotionality (Carstensen et al., 1999; Carstensen, 2021). Time horizons influence goal priorities and emotional experience. Specifically, growing mindfulness of their mortality may lead older adults to pursue emotionally meaningful goals by investing psychological and social resources to optimize their emotional well-being and to maintain emotional significant relationships (Burr et al., 2021; Carstensen, 2021). When facing conflicts, older people tend to focus on enhancing their relationship, rather than on winning an argument (Birditt et al., 2005).

Moreover, older couples — in contrast to younger couples — are likely to use passive coping strategies, such as waiting to see if things improve on their own and avoiding quarrels in order to not escalate the conflicts (Charles et al., 2009; Luong et al., 2011). Nonetheless, according to the theoretical model of strength and vulnerability integration (SAVI), if there is a conflict that cannot be avoided or addressed by accumulated regulation strategies (e.g., the capacity to adapt to loss), the conflict is more demanding than at younger age due to the reduced physiological flexibility to handle high levels of sustained emotional arousal (Charles, 2010). These developmental changes suggest that age modulates coping strategies and subsequently the perceived seriousness of conflicts.

Although this may suggest that there is less relationship conflicts in older age, data on divorce rates show that the amount of gray divorce is increasing. These successful conflict management strategies are mainly related to those conflict situations that can be avoided. However, the aforementioned SAVI theory also describes how much more difficult those unavoidable conflicts are for older people. This leads researchers to investigate which conflicts are specifically involved and which of them are age-specific (i.e., typical for older couples) in order to better understand the grey divorce phenomenon.

Lin et al. (2016) specifically tested the assumption that couples wait to divorce once the children are grown and have left the household (i.e., an empty nest may explain relationship dissolution; Bair, 2013). However, they found no support for this assertion. With the use of a life-long perspective, other prior studies have also examined the role of transitions (e.g., empty nest and retirement syndromes) in relationship stability. Although these moments may put less demand on the alignment of family and work life, the couple deals with spending more time together (Hatch and Bulcroft, 2004) and the study of Lin et al. (2016) suggests that many dissatisfied couples may see no reason to delay divorce until their nest is empty.

Similarly, both Karraker and Latham (2015) and Lin et al. (2016) examined the relationship between the onset of physical illness and the risk of divorce among middle-aged and older adults. Only the onset of illness for a wife was associated with an elevated risk of divorce (Karraker and Latham, 2015). Although these findings were not robust, accepting the role of a caregiver in a relationship may strain the relationship. Caring about a partner often has a moral dilemma for the determination of when it is permissible to leave a partner and under what conditions. Specifically, caring for a sick partner in a long-term partnership is often perceived as a commitment or obligation that the partner fulfills without really having a choice, while in re-partnered couples it is perceived as a voluntary decision (Koren, 2011).

Likewise, retirement is discussed in relation to relationship quality (Szinovacz, 1996; Moen et al., 2001). Not all older people, mainly those with good health conditions and having a working spouse manage to adapt to retirement well (Kim and Moen, 2002; Barbosa et al., 2016). Some experience symptoms of depression. Tension builds in their relationships, especially with their partner (Cooley and Adorno, 2016). Issues might arise due to traditional gender divisions of labor within marriages but also in older couples who consider re-partnering in later life (Upton-Davis, 2015).

Some challenges regard changes in personality. On one hand, studies show a positive shift toward increased friendliness and reduced extraversion in later life (Field and Millsap, 1991; Berg and Johansson, 2014), thanks to which the readiness to engage in conflict may be lowered (Iveniuk et al., 2014). On the other hand, Maiden et al. (2003) suggest that unfavorable life situations, such as illness, may lead to an increase in neuroticism and a decrease in extraversion. Therefore, people in later life may encounter attitudes and behaviors in their partner they are not accustomed to, which could present new challenges.

Nonetheless, some problems in later-life relationships may resemble problems at a younger age. Infidelity, for example, is ever present as a reason for divorce along with incompatibility, drinking or drug use (Amato and Previti, 2003) and growing apart (Amato and Previti, 2003; Hawkins et al., 2012). According to the analysis of General Social Survey data (United States), affairs in spouses over the age of 60 were increasing in the past decades. In the 15-year span from 1991 to 2006, the lifetime rate of infidelity for men over 60 increased from 20% in 1991 to 28% in 2006. For women over 60, the rate of infidelity went up from 5% in 1991 to 15% in 2006 (Parker-Pope, 2008). In the analysis of more recent GSS data, Wolfinger (2017) has found that by 2016, 20% of older respondents indicated that their marriages were nominally adulterous, compared to 14% for people under 55, and that these people are mostly in relationships of 20 to 30 years. Along with the higher rate of cheating, relationship dissolution is more common among those who reported extramarital sex (OR = 2.7/3.9 for divorced/separated persons), which points to its potential destructive effect on long-term relationships (Djamba and Kimuna, 2020).

Having partner difficulties at older age may not seem common and acceptable by society. Some topics may be too sensitive to share (e.g., disagreements or difficulties in sexual life) such that older adults might be concerned about using a traditional venue for handling their problems. This would lead them to use online counselling to ask for help or tailored advice (Powell et al., 2011; Moorhead et al., 2013). At present, a significant percentage of older (55+) individuals are active internet users (CZSO, 2022) and online counselling services are widely available. They offer anonymity and a less stigmatized setting for consultations, which may be desirable in this area of concern. They are also available for those who cannot turn elsewhere (Powell et al., 2011; Lee and Lin, 2020). Moreover, some researchers view the internet as especially important with respect to sensitive issues, since the anonymity — a facilitator of disinhibited communication — may expand the opportunities to discuss sex-related and other sensitive issues (Cooper, 1998; Ross, 2005).

### 1.1. Research aim

Despite the growing body of research on the specifics of later-life relationships, there is still a paucity of studies systematically analyzing relationship difficulties and strains at older age (60+). Moreover, little is known about the concerns with which a continuously growing population of internet users turn to online counselling websites to disclose sensitive and taboo issues (Faverio, 2022). The focus of this study is, in addition, highly prompted by the rise of grey divorce. In this respect, we aim to gain an insight into the strains and stressor patterns in the relationships of older couples by conducting a thematic analysis of later-life relationship-related queries that were posted on counselling websites, by asking:

- RQ1: What types of problems do older adults experience in their relationships?
- RQ2: In what ways does age influence the relationship problems that older adults encounter?

## 2. Methods

### 2.1. Sample and procedure

This qualitative study was conducted in 2020. It was based on the analysis of publicly available queries that were posted on professional counselling websites that are maintained in the Czech language. The websites did not require registration for access. Most of the queries were not dated. Among those that were included, they ranged from 2004 to 2020. Nine queries were posted before 2010. As for our positionality, the data were coded by researchers in young and middle adulthood. The research has been conducted in the Czech Republic, which is racially and ethnically homogeneous and which, like other European countries, has a relatively large percentage of older citizens (approximately 20%; CZSO, 2021). The Czech Republic is known to be one of the most secular countries in Europe with less conservative views towards family, marriage, and children (Chromková Manea and Rabušic, 2021). This can be attributed to the country’s communist past, which led to a focus on secularization, the liberation of abortion and divorce law, and pro-family policies (e.g., providing loans to young families or emphasizing the importance of quality sexual partnerships; Lišková, 2018; Bělehradová and Lišková, 2021).

Prior to the research, we used Google and Czech browsers (e.g., Seznam.cz) to create a list of counselling websites with the following criteria: they were available to Czech-speaking internet users; they were run by either professionals (e.g., psychologists, psychotherapists, sexologists, and other specialists, like urologists, gynecologists) or non-professionals, such as relationship coaches; and they had content that was made public (i.e., publicly available to any internet user). The majority of the websites were focused on psychological (13), sexological (six), medical (four), and urological (one) themes. Upon the selection of the counselling websites, we conducted a pilot study to develop more nuanced sampling criteria in order to include all of the relevant queries. With respect to our focus on later-life relationships and the associated problems, we determined the following criteria for the selected queries: (1) they concerned themes and problems related to partnerships; (2) the query authors dealt with issues that concerned their own relationship; and (3) the discussed issues concerned couples in which at least one partner was aged 60 or over. This bottom-age limit corresponds to the onset of older adulthood (WHO, 2020). For queries that lacked the age of the partners, we sampled queries in which there was a stated relationship length that was at least 40 years long. The criterion of the relationship length (i.e., at least 40 years) was derived from data provided by the Czech Statistical Office. According to these data, the mean ages for first marriages for brides and grooms were 21.6 and 23.9, respectively, between 1964 and 1990 (CZSO, 2006). To meet the third criterion, at least one of the age criteria had to be met (age of the poster; age of the partner; length of the relationship).

**Figure 1:**
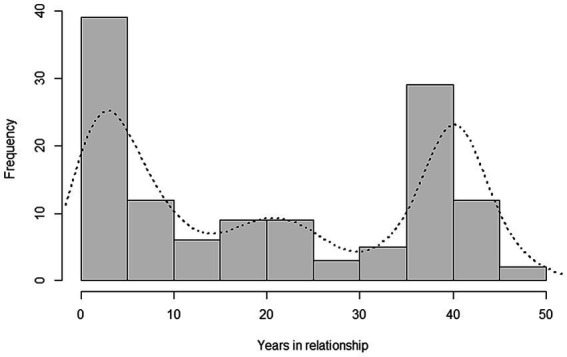
Length of relationship histogram [*n* = 126 (persons; 56%)].

Our final sample consists of 225 queries (see Table 1). The data are available at: https://osf.io/akfjb/ or by emailing the corresponding author. The majority of queries met age criterion 2 (partner 60+, *n* = 145), with age criterion 1 (poster 60+, *n* = 95) following, and age criterion 3 (length of relationship, *n* = 35) being the least met. For those posters who provided the information, their age ranged from 26 to 77 (*M* = 57.3, *SD* = 9.5, *n* = 164 (73%)) and the age of their partner ranged from 39 to 88 (*M* = 62.8, *SD* = 6.2, *n* = 154 (68%)). On average, there was an age difference of 10.2 years between the partners (*SD* = 9.1, Median = 7, range = 0–40, *n* = 115 (51%)), but the distribution of these differences was heavily positively skewed. Two posters did not indicate their gender, but the rest of the sample was predominantly female (*n* = 178 (80%)). Only one poster indicated non-heterosexual orientation. Three queries did not provide the information about the gender composition of the couple. This allows us to assume that most couples consisted of a male and a female and the posted problems pertained mainly to heterosexual relationships. Out of 181 (80%) posters who provided the information, 148 (82%) lived with their partner and 27 (15%) were not cohabiting, but considered themselves to be living with their partner. The remaining six posters (3%) did not consider themselves to be living with their partner at that moment. The length of the relationships were apparently bimodal (see Figure 1). Our sample included posters who were in relatively short-term relationships (in this context, 0–10 years) and long-term relationships (here, 35–45 years). To sum up, in most cases, our posters were approximately 60 years old, mostly female, and they asked for advice for relationship problems with their heterosexual partner, with whom they cohabited, and who was not more than 15 years older or younger than themselves.

**Table 1:** Final sample (*N* = 225) that met the selection criteria.

Poster is 60+ years old	Partner is 60+ years old	Relationship lasted 40+ years	Count	%
x			49	22
x	x		35	16
x		x	7	3
x	x	x	4	2
	x		106	47
	x	x	2	1
		x	22	10

The columns represent three criteria for selecting the sample. ‘x’ indicates which criterion the respondent’s contribution fulfilled. Some contributions met multiple criteria.

The study was approved by the Institutional Review Board of the authors’ university.

### 2.2. Data analysis

The queries were coded for socio-demographic characteristics. A qualitative analysis was conducted using a thematic analysis, specifically an inductive analysis (Braun and Clarke, 2006). This method provides a flexible and useful research tool to identify, analyze, and report patterns (i.e., themes) within the data. The inductive approach allows for the creation of codes and themes that are derived from the content (Braun and Clarke, 2006).

At the beginning of the analysis, the researchers 1 and 2 went through all the queries to get familiar with the data. During this process, they assigned initial codes to summarize the main issues in each query. Next, they looked for recurring themes across the data and used different colors to represent these themes for better organization. The researchers 1 and 2 discussed the preliminary themes together, assessing whether certain themes were distinct or possibly related, and whether the theme names accurately captured the patterns found in the data. They also made sure that the assigned codes matched the concerns expressed by the posters.

In the following phase, the researchers defined and named the themes in a way that reflected their essence. Specifically, they analyzed how age was expressed in the queries, noting any references to specific age groups or age-related concerns. The researchers used these findings to derive three categories that reflected the content and meaning of the queries and responded to the question of how the types of problems interact with the influences of the age.

Finally, they compiled a report of their analysis, including excerpts from the queries to effectively illustrate the themes they are presenting.

## 3. Results

Using inductive thematic analysis, we identified four themes for the problems discussed on the online counselling platforms: (1) jealousy (or the unwelcome presence of other people in the relationship, *n* = 79); (2) relationship estrangement and cooling (*n* = 44); (3) undesirable changes in personality (*n* = 33); and (4) illness (*n* = 53). Queries that did not fit into any of the themes (*n* = 16) were mostly about not being sure if to start a new relationship with somebody they already knew, about age differences, and about finances. Since there were only a few queries for each theme, we did not create separate categories for them.

We also identified three themes that made the problems specific to older age or more demanding in later life. These are (i) the lack of norms for relationships at older age; (ii) the absence of resources to tackle the issues; and (iii) the personal accounting of the time spent and the time remaining.

### 3.1. Infidelity and jealousy

This category captures relationship problems linked to the presence of a third person (e.g., an extra-relationship partner, ex-partners, a child from a previous marriage). Infidelity, by either partner, dominated this cluster of discussed issues. The incidence of cheating or jealousy was present in a large portion of enquiries. The category dominated our sample. The loss of trust and the threat to relationship stability was perceived as great by the posters: *“I am 64 years old. Last week I read a love SMS addressed to my husband and since then I have had a mental breakdown. We have been together for 42 years without the slightest problem. I love my husband and I do not want to lose him”* (F05, female aged 64). This extract is typical for this category – the surprise of having to face infidelity at an advanced age and after so many years together.

If the affair had ended, posters turned to online counselors to discuss the issue of restoring trust in their life-long partner. The renewal of trust was important due to the fact that the posters still loved their partner: *“I still love him. I cannot imagine life without him and I do not want to be alone at the age of 60. … I feel very stupid … and I do not trust him. Of course, he claims he does not have anyone, but he said that even though he had (someone) … I do not want to be the idiot I’ve been for so many years any longer. I’m afraid something will come again and I do not know how I should handle it”* (S05, female, 60). In this case, the betrayal of trust seemed to be worse because of the amount of time that they woman had been cheated on. She also emphasized her worries about the loss of her relationship at this age.

Jealousy also concerned people from the past, such as an ex-partner, or children from a previous marriage. Some posters mentioned their partner’s intolerant behavior towards their children and grandchildren: *“My partner hates my children and grandchildren, refuses to communicate with them and does not answer their greetings. … When I see my children, grandchildren, he starts to be mean and vulgar to me. He does not speak to me for days, blames everything (on me). How do I solve this?”* (G20, female, 60).

### 3.2. Relationship estrangement and cooling

Another cluster of relationship problems concerns between-partner estrangement and a lack of warmth within the couple that was manifested by negativism, a loss of common activities, or the lack of interest in the partner or partnered sex. Many posters mentioned an issue of becoming *chilly* towards each other in the relationship:

*“I want to touch her. She dodges. It bothers me a lot… I try to be gentle but after hearing ‘What do you want, again? … We’re tired of the relationship. She’s very tidy, capable, but her behavior to me is destroying me. I’m taking meds for my nerves. I thought it would be better in retirement, but no”* (S17, male, 60). This case points to the potentially negative impact of the perceived lack of warmth in the relationship on the poster’s well-being. This estrangement usually appeared in various domains of later-life relationships, including partnered sex. Some posters described how they absolutely lost the sense of partnership: *“We have not slept together for the last three years. There is nothing to communicate about at home. At work it is just the necessary work communication. We can no longer live together … I would like him to move out, but I feel that I owe him… I can no longer communicate with him. We do nothing together. We both go home whenever we want. We do not say anything”* (F08_1, female, 44). The repetitive motive was negativity and the descriptions of the things that were missing, that “are not.”

Other posters used the term “allergy” to describe their experience towards the partner or vice versa. This problem appeared in long-term couples and in newly formed relationships. The latter case was treated as less understandable for posters: *“The whole thing is tied to a certain irritability of a partner who was a very nice and fun companion a year ago. ‘Today’ he is just and above all either quiet — he does not talk — or he points out my deficits or lashes out because of little things. It seems to me that he is allergic to me”* (F25_1, female, 48).

In the case of newly established relationships, between-partner estrangement took a specific form in which the relationship is reduced to “cheap labor force.” Posters shared their impressions of investing much more of their time and money into the relationship than their partner. In a majority of the cases, this concerned female posters: *“I still work at his house and garden. I even maintain the grave of his wife. I’m actually cheap labor for him. I think he cannot love me at all or that he’s not normal”* (F16_1, female, 56).

### 3.3. Undesirable changes in personality

Many posters wrote about difficulties regarding their partner’s temper and behavior because it negatively affected the relationship and manifested via aggressiveness, laziness, stinginess, and sourness: *“My husband can constantly make himself mad and add stress. But not only for us. Lately he has been verbally attacking complete strangers about petty things. He said he won‘t control himself”* (F06_1, female, 40+). These changes ranged from a sudden shift to a gradual accentuation of negative traits that the partner had always had but which became more pronounced with age: *“Somehow I cannot explain the transformation of a man who was reliable, kind, and loved his family, then suddenly turned into a philanderer (mildly said)*. *Today he is 60 and he’s someone else”* (F09_1, female, age N/A). In another case: *“He is an extreme choleric. He was always domineering and moody, but now, in old age, it has multiplied several times”* (S14, female, 60+).

Posters provided insight into the context of personality changes within their relationships. Some of them mentioned retirement as a negative turning point. Specifically, they elaborated on the increase of time spent together, which highlighted the negative aspects: *“My husband and I have been together for 43 years. Everything was normal when we were working. Now we are both retired and we have a bit of cabin fever. I’m communicative. My husband is not … I do not understand how some men at his age can change so much. Otherwise, he is very kind. He does not even have friends, because he is not talkative. I try to entertain him, but I always get a brief answer. I like him, but I do not know what to do”* (S12, female, 63).

Although this enquiry contains the lack of mutuality that was typical for the examples in the *Relationship estrangement and cooling* category, that did not occur in this case. Rather, the gradual loss of things in common and the loss of interest in the partner were due to personality characteristics that had always been present but that had become more pronounced. The two categories can be distinguished based on the types of descriptions used by the inquirers. The *Relationship estrangement and cooling* category contained descriptions of the general deterioration of the relationship, while the *Undesirable changes in personality* category described specific changes in the partner’s character traits.

### 3.4. Illnesses and somatic issues

The last category included queries that jointly discussed the negative effects of illness on the relationship. It consisted of a wide range of topics: providing care; the end of the partnership and the start of caregiving; sexual difficulties and their impact on relationship satisfaction; and mental illnesses. The problems in these queries were not only somatic but, in some way, interfered with the functioning of the relationship.

Most of the queries concerned sexual dysfunctions or care-related issues. Although the topic of sexual life was introduced in the *Relationship estrangement and cooling* category, both posters and partners in this category were specific about the sexual problems that motivated them to improve their sexual lives, usually with a new partner: *“Due to my wife’s illness, I did not need to deal with my weak erection for a long time. She died two years ago after 56 years of cohabitation. Now I have a girlfriend a year younger than me and I think it would be worthwhile to solve my erection problems”* (F35, male, 74).

Other related enquiries concerned the posters themselves, specifically how much they ought to help without regard to their own well-being and, eventually, how to leave the relationship completely. This appeared more often in queries where a discrepancy in the age of the partners was present. Posters viewed themselves as too young to be spending their lives as a care-giver and expressed their desire for a new relationship that would fulfill their needs. But since their partner was dependent on them, they struggled with a guilty conscience that prevented them from leaving:

“(I feel) emotional blackmail from my boyfriend when I mention that I can no longer be with him, that I need a man with whom I would be with out of love and not compassion. I want to leave him. This relationship is gradually killing me… Sex with my boyfriend humiliates me. Please, do I have the right to leave a man who is older, sick? What will my conscience do to me if I have left a man who loves me so much? I always tell him that I will be happy to help him even after we break up, that I just want him to let me leave him, but he does not want to” (S11_1, female, 48).

Part of the illness-related queries were about the suspicion of mental illness. The posters asked if the signs they observed in their partners were clinically significant and how they should convince their partners to ask for a professional help:

“It’s about the pathological jealousy of my cancer-affected husband. We are both already old. My husband is 67 years old and I am 62. We have lived together for over 40 years… His moods change every 10 min… He has even physically attacked me… then in the next moment he caresses me, kisses me like nothing happened… [it goes from] extreme to extreme. I want him to visit an expert together or on his own! HE REFUSES!” (P38, female, 62).

In this case, the poster turned to the internet to face the partner’s aggression and when other previous attempts to solve their problems have proved to be ineffective.

### 3.5. Influences of age

Some of the issues mentioned above are more likely to happen to older couples, such as illnesses and the increase in time spent together. The rest of the above-described problem clusters may appear in middle age or earlier, but there are age related influences that make them different and more demanding when handled in older age. The analysis identified three overarching themes: (i) the lack of norms; (ii) the lack of resources for tackling the problems; and (iii) the personal accounting of time spent and time remaining.

#### 3.5.1. Lack of norms

Some posters turned to online counselors because they were not sure about what was normal for ageing, sexual life, and later-life relationship troubles. They lacked norms to which they could refer in order to better understand their situation. Their queries were largely about their unpreparedness for tackling the problems that they did not expect at their age or that may have emerged in later life. This issue specifically concerned changes in temper in older age. The posters wanted an expert opinion to provide insight into their situation and tell them what to do. Quote by F09_1, female, 60+ (see above) illustrated how the change of a partner’s behavior was so unexpected that the wife searched online for insight.

Many queries were about what was normal for later-life relationships. This concerned infidelity at older age. Some posters were not sure what is “normal” or acceptable. Those facing such a situation admitted that they did not anticipate the relationship problem at their age and wondered whether it happened to others as well: *“Is it normal? I want to understand it as a person, because I am ashamed in front of my children and our friends…. Am I more of an exception or is it normal nowadays to ‘throw away’ old wives when their husband gets luck and a young lady falls in love with him, as my husband told me?”* (S22, female, 60+). The poster genuinely did not expect that infidelity would endanger their relationship at their age. She acknowledged that she experienced a loss of security in her life due to the presence of the extramarital relationship. She desired to know whether this happened in other relationships to better understand her personal situation.

In addition, some enquiries mentioned taking into consideration social norms, such as the social acceptability of divorce and leaving the family at such a late age: *“At this age to divorce and divide property? I would have died of shame if only because of my adult daughter”* (S04, female, 63). In her view, divorce in later life was not socially acceptable. Another aspect of questioning the right to leave the family was discussed by younger posters who were in a relationship with an older partner. They deliberated on whether it is appropriate to leave a person that can be no longer a partner for them due to health problems and who has become reliant on their care.

Social norms or worries about the opinion of others also concerned sexual life, such as the level of sexual appetency and the fear of being pregnant in the 70s, since the inquirers were too shy to discuss these topics offline.

#### 3.5.2. Lack of resources to tackle the issue

A large part of the posters mentioned how difficult it was to tackle relationship problems and leave their relationship due to their lack of resources. In the analysis, we identified three types of resources that were lacking: finances, social support, and personal resources that diminished with age (e.g., mental and physical strength, health, attractiveness).

Many posters mentioned their lack of finances as a circumstance that made their relationship problems worse. Without these resources, it was much more difficult for them to leave the malfunctioning relationship and find a new one or to manage the current divorce or breakup. *“Please advise what I should do when my husband has acquired a mistress after 44 years of marriage…. It hurts a lot. I do not know whether to solve it with a divorce. Our money is in real estate. It’s hard to pay for housing. After years of saving and working hard, I’ll leave with a bare ass”* (S15, female, 60+).

Other posters mentioned the lack of social support, like children leaving the home and the passing of their parents: *“After the children left home, I had the desire to improve my life again … the children are far away and, of course, they do not want to come home. Trying to solve it with infidelity is not my style, but I am abandoned and often slightly depressed. I miss people. We live in solitude in the woods, I do not have a driver’s license and I’m not in a position to make friends in a village eight kilometers away … I adopted a stray cat a few years ago. I’m talking to her, but it’s kind of not enough”* (F19, female, 60+). Other posters felt neglected by their partners but they seemed to have some other social source that helped them cope. Now, when the support is gone, it is harder to handle their malfunctioning relationship.

Other posters perceived the loss of youth as a diminished personal resource. They realized that their youth had some advantages, such as physical attractiveness, which they might not have anymore: *“I still love him, but I always remember that he was with her and I think I’m completely useless here and that I‘m too old to have a chance with someone better”* (F36_1, female, 55). In this case, the impact of the partner’s infidelity could be even more painful when she started to think about her reduced chance of finding a better relationship at her age. They mentioned the lack of unspecified strengths and energy to face and resolve such problems, connecting this to their age: *“I wanted to get a divorce a long time ago. Now, after taking care of my husband after surgery and illness, I feel old and tired. Even though I thought I would be calmer and happier without him, I’m almost at the bottom. I often cry. Everything suddenly feels like waiting for death. I have no idea how I will deal with this at my age”* (F13, female, 60+). Physical limits factor into her management of the amount of stress and the assessment that, earlier in her life, it would not have been so demanding on her.

#### 3.5.3. Personal accounting of time spent and time remaining

We also identified that the perception of time played a role in the assessment of the gravity of relationship problems. There were two directions for time perception – time spent and time remaining. The former was related to the loss of long-term investment. The latter was linked to poor prospects.

#### 3.5.4. Time spent

Many posters emphasized the time spent in relation with their investment into the relationship. The loss of the relationship they were building for 40 or more years was that much more difficult because of everything that they had put into it and the sheer amount of time they had spent in it: *“I have been married for 40 years, a satisfied, unwithered relationship… A week ago, he had a semi-social event. I went for a walk and saw him, a few dozen meters from our house, walking hand in hand with a lady. It was a complete shock to me. He had my absolute confidence… I feel like I‘m only giving and the confidence that I had in my husband is gone”* (P32, female, 60+). Late-life infidelity proved to be a strain for the posters because they felt forced to reevaluate many years of shared time: *“My husband forgot his cell phone at home next to the computer and I read three text messages, from which I concluded that I do not live with a man who is not interested in sex, but rather with a homosexual…. I know I have to help myself to live on. I know that. But the feeling of living with someone other than who I had thought for 40 years is quite devastating”* (F02, female, 60+). Time spent in the relationship was a factor that made the perception of relationship problems more severe.

#### 3.5.5. Time remaining

Problems in later life are more demanding because romantic partners realize that, if something is not going to happen, the problem will last till the end of their lives. Had they more of their life ahead of them, they might have hoped that the problem would somehow solve itself. But the prospect of a short time frame made them think twice about the problem and its solution. This contributed to their dissatisfaction and probably enhanced their help-seeking behavior: *“So the situation is that I do not know when my wife is telling the truth or lying. I’m paying off debts and I do not know what to do. This situation has persisted for several years. I am 62 years old and the idea that I will be responsible for her behavior and debts until my death does not add much enthusiasm to my life”* (F16, male aged 60+). In a way, some queries treated the time remaining as a resource of its own that needed to be spent wisely.

The time they had left also gave rise to questions of whether it was still worth it and whether it is worth changing something at their age. In the previous case, the realization of the time remaining stimulated the change, but, in some cases, it might have inhibited it. The realization of the lack of time remaining made them think twice about such an important decision, like whether to stay in a relationship: *“I’m not sure if this relationship, for the few remaining years of our lives, is meaningful”* (G23, female aged 60+). In some queries, posters asked if it is worth risking the change: *“My husband and I have lived together for 42 years. Sometimes it was fine and sometimes really bad. Lately, however, it’s starting to get dramatic. He’s clawing into me so hard … and he goes to extremes… Is it worth trying to solve something at my age, and how?”* (S13, female aged 60+). In other cases, the time remaining also meant that it was too late to make fundamental changes: *“He actually left me in ignorance for eight years and thus deprived me of the opportunity to reorganize my life”* (P37, female aged 61). The dishonest communication led to the poster to have less time left to reorganize her life. From the query, it seems she was aware of the fact that the time remaining was precious and that there was a need to be economical.

## 4. Discussion

This study aimed to gain insight into the strains and stressor patterns in the relationships that older adults encounter and post about on online counselling websites. The findings showed that (i) the types of problems themselves (e.g., infidelity, cooling of the relationship, illness, undesirable change of personality) were not unique and, according to prior research (Gravningen et al., 2017) they were similar to relationship issues common for younger age categories and that (ii) the way the posters processed and perceived the problems as pressing was influenced by specific aspects of ageing, namely the norms and expectations related to age, the resources they could use to cope with the problems, and the perspective of time.

Infidelity, jealousy, and the unwanted presence of a third party at older age was the largest category in our study. Our findings suggest that this theme does not cease to be relevant in older age. This resonates with prior research (Amato and Previti, 2003; Wolfinger, 2017), according to which infidelity in later life is still present and on the rise. Although it was previously thought that the reason for the increase of grey divorce lies in the challenges of the “empty nest” (Bair, 2013), our study has shown that jealousy and infidelity were the predominant reasons for distress and dissatisfaction in relationships and for the consideration of a divorce.

Posters talked about changes in their partner’s personality. Meaning, the writers noticed changes in their partners’ behavior, thinking, or communication, and they labeled it as a shift in personality due to the magnitude of this change. Research shows a shift with age towards higher extraversion and agreeableness (Oltmanns et al., 2020), but only in connection with other variables, such as the state of executive functions (Williams et al., 2010). The changes in personality that the respondents perceived could be related to the deterioration of executive functions and possible mental illness. Some changes in behavior that might lead to social inappropriateness and lesser empathy may be an early indicator of dementia (Desmarais et al., 2018). For other respondents, however, it may not have been a change in the partner’s personality, but rather a change in the setting. In retirement, the couple begins to spend more time together, and it is possible that they began to perceive their personalities differently and without adjustment to their different life roles. The design of our study does not allow us to provide an answer for the cause of these problems. This is both a limitation and a possible direction for further studies.

Similarly, the theme of relationship estrangement and cooling was viewed as an issue that made participants unhappy with their relationship. This type of relationship problems may bear some resemblance to “growing apart” that has been describe in prior research using a life course perspective and that points to incompatibility as a result of age at marriage (Amato and Previti, 2003; Hawkins et al., 2012). However, incompatibility presented only one aspect of our theme that in addition included a lack of warm and care.

Our results show that time remaining substantially shaped the perception of the problem and the possible solution. Although limited time obviously helps people to set priorities that result in the pursuit of emotional goals, as suggested by the Socioemotional Selectivity theory of Carstensen et al. (1999), our study showed that acknowledging the time remaining may make relationship problems and relationship dissolution more stressful and painful. Time remaining intensified the perception that the prospects had dramatically shrunk. Due to a decrease in the time remaining, there is, from the perspective of the participants, a lower chance to find a new meaningful relationship and lesser space to start anew.

The impending inevitability of approaching mortality provides not only a different perspective but can also create anxiety that people need to cope with (Greenberg et al., 1986). According to Terror Management Theory, people choose various ways to deal with this anxiety, and one of these ways is through their functioning in relationships. However, the manner in which this occurs is moderated by several factors, including gender, attachment, and self-esteem. Based on these specific moderators the relationship phenomenon such as rejection sensitivity, desire for multiple sexual partners, intimacy needs, and jealousy are either activated or inhibited (Plusnin et al., 2018). Previous research has shown that inhibition of potentially harmful relationship is more common among securely attached individuals, females, and those whose self-worth is not contingent on potentially harmful behavioral intentions, such as the desire for multiple sexual partners. However, males, individuals with anxious-insecure attachment styles, and those who rely on relational self-esteem enhancement to cope with mortality, are more likely to engage in potentially harmful behaviors after mortality salience (Plusnin et al., 2018).

The presence of multiple influencing factors, such as attachment, on how perceptions of mortality affect relationship dynamics may explain why, despite both partners being in a similar position with diminishing resources and limited time remaining, one may decide to jeopardize the relationship through infidelity, as seen in many of the queries in our study. This apparent paradox of infidelity can be explained by the fact that individuals with insecure and anxious attachment styles often have a greater fear of being alone. Consequently, they may be more prone to infidelity, as they are more afraid of being single and choose a ‘bet-hedging strategy’ in their relationships (Sakman et al., 2021).

Similarly, the perception of time already spent in later-life relationships was present in the queries. Posters used this information to emphasize that the problem had been present for a long time or that they were having a crisis after such a long time spent in the relationship. They voiced their frustration for investing so much into the relationship and learning that they received little and that they would lose everything due to a risk of relationship dissolution. If we take into account the perspective of the Investment model (Rusbult, 1983) — according to which relationship satisfaction is a function of cost, rewards, and the comparison of alternatives — then the investment of their whole adult life into something they could lose, or have already lost, might suggests that they can no longer rely on what they had accumulated throughout their lives. Using the perspective of the strength and vulnerability integration model (Charles, 2010), some posters might have felt that their lifelong experience with their capacity to deal with difficult situations was shaken.

Acknowledging these time perspectives, some posters conveyed the feeling of *entrapment,* where the rewards in their current relationship were low but the alternatives, and particularly their resources, were limited (e.g., perceived perception of fewer possible partners, no longer feeling attractive). Being entrapped may be accentuated in later-life relationships due to age-related work inactivity and the growing inaccessibility of older partners in the dating/marriage market (Span, 2016). In this respect, the findings suggest that older adult’s vulnerability and their likelihood to stay in an unsatisfactory relationship may increase because of the resources that are depleted by age.

Queries often concerned the norms of relationship and sexual functioning. Shame probably hindered communication with doctors and partners. In some cases, they explicitly acknowledged this. The anonymous space of the internet provided an option to address it more openly since the internet is known for facilitating the communication of taboo issues (see Hinchliff et al., 2021; Ševčíková et al., 2023). As for the lack of these specific norms, it is also possible to assume that the tendency to search for them may derive from generational changes. With each cohort, the life expectancy extends and results in more older people who enter into later life as a couple and then experience a substantial part of their later life as a couple (CZSO, 2018; WHO, 2022). This might give rise to a new generational experience for which norms are lacking, resulting in an increased demand to get oriented in later-life relationship challenges that previous generations could not have experienced.

Regarding the limits of our study, even though the number of older internet users is continually increasing, there remains a substantial portion who do not use the internet at all or who do not feel comfortable using technology in the same manner as our posters. This leaves unknown differences between users and non-users related to intimate relationship. Therefore, our findings are limited to the population of internet users, mainly to female users who appear to be open to addressing their problems online. They may benefit from the anonymity offered by counseling platforms to discuss specific topics, such as a perceived sense of failure in their lives. It is worth mentioning that the disproportionate prevalence of women in seeking help has been observed in prior research (Revenson et al., 2016; Ševčíková et al., 2023). In addition to non-users of the internet, those who were not present in our study can be those who have been identified and already cared for by the healthcare system. This could explain why our sample has only a few questions about serious health complications and many questions about intimate life and health. On the contrary, the selection could have captured and highlighted the problem of missing norms followed by the difficulty to communicate some topics, such as infidelity, leaving an old partner, and the shame associated with them. Our sample included queries from posters who were in relationships with significantly younger or older partners. The issue of substantial age differences affected themes that emerged the analyzed posts (see for instance illnesses, somatic issues, or the absence of norms). Another limit of our study concerned the impossibility of asking the posters for more details and reasons for the identified relationship problems. We based our study on their own formulations and conceptualizations, which were authentic, but at the same time might have been limited by their ability to describe underlying or inconspicuous details in more depth. Lastly, it is important to bear in mind that the sample included queries from posters of different cohorts who might be dealing with various issues. This distinction is particularly evident in the study sample, where both long-term relationship issues and problems in recently established relationships emerged. This suggests that younger generations could be more open to forming new partnerships later in life compared to representatives of older birth cohorts. To address the limitations of the current study, future research could consider utilizing a more randomized sampling approach, rather than relying on self-selected participants. This would enhance the representativeness of the sample and minimize the potential impact of self-selection bias. Additionally, it would be beneficial to examine reasons of potential relationship issues. It remains unclear whether elevated stress levels in later life hinder the socio-emotional expertise and problem-solving abilities that older individuals have developed over the course of their lives. In simpler terms, high levels of stress may temporarily distort the partners’ ability to empathize, resulting in disrupted emotional communication within the couple and, at times, leading to temporary aggressive behavior and emotional detachment between partners. By adopting these strategies, future studies could provide a more robust and comprehensive understanding of the issues faced by couples in relationships, and contribute to the development of effective interventions and support services.

## 5. Conclusion

This study showed that relationship problems discussed by older adults via online counselling were not unique compared to the relationship issues that are common for younger age categories. The way the posters processed and perceived their problems as pressing was influenced by their views that their accumulated experiences and resources were shaken and much less could be renewed due to their remaining time. This view may challenge older adults’ physiological flexibility, resulting in difficulties with handling high levels of sustained emotional arousal. These may have implications for clinicians, who should carefully reassess their resources to renew older adults’ ability to adapt to loss.

Our findings can provide practical benefits by enhancing the comprehension of the challenges that older individuals may encounter in their romantic relationships. Increased awareness can prompt practitioners to inquire more about specific areas, particularly on sensitive topics, such as sexual functioning, and facilitate cooperation with their clients. Furthermore, our results indicate that financial constraints may result in older individuals remaining in a relationship even if it is no longer fulfilling. This highlights the impact of socioeconomic and social statuses on the well-being of older adults and emphasizes the difficulties they may face in resolving such issues.

## Data availability statement

The datasets presented in this study can be found in online repositories. The names of the repository/repositories and accession number(s) can be found at: https://osf.io/akfjb/.

## Ethics statement

The studies involving humans were approved by the Research Ethics Committee (REC) of Masaryk University. The studies were conducted in accordance with the local legislation and institutional requirements. Written informed consent for participation was not required from the participants or the participants’ legal guardians/next of kin because we have collected publicly available data posted online by individuals themselves, with their understanding that the queries would be publicly accessible.

## Author contributions

SS reviewed all of the queries and identified common themes and motifs. Throughout this process, AŠ provided ongoing supervision and feedback. SS and AŠ finalized the labels for these categories and wrote the majority of this paper. JG provided statistical analysis and wrote the parts of the paper concerning them. All authors contributed to the article and approved the submitted version.
